# Supplementary material for: Comparative genomics shows that viral integrations are abundant and express piRNAs in the arboviral vectors Aedes aegypti and Aedes albopictus
Source: BMC Genomics. 2017 Jul 5;18:512. doi: 10.1186/s12864-017-3903-3 (PMC5497376; doi:10.1186/s12864-017-3903-3)
Supplement: Supplementary file 4 — DNA-seq library statistics. Total number of reads, percentage of mapped reads and percentage of properly paired reads from DNA-seq of 16 individual Ae. albopictus mosquitoes (Foshan strain). (DOCX 20 kb) [file 12864_2017_3903_MOESM4_ESM.docx]

**Additional File 4: Table S4. DNA-seq library statistics**. Total number of reads, percentage of mapped reads and percentage of properly paired reads from DNA-seq of 16 individual *Ae. albopictus* mosquitoes (Foshan strain).

| Libraries | Total reads | Mapped reads | % mapped reads | % properly paired reads |
| --- | --- | --- | --- | --- |
| 1 | 211,039,265 | 203,261,786 | 96.31 | 74.56 |
| 2 | 229,321,960 | 223,161,305 | 97.31 | 80.06 |
| 3 | 296,165,976 | 286,962,998 | 96.89 | 80.04 |
| 4 | 238,158,053 | 230,796,204 | 96.91 | 78.49 |
| 5 | 211,746,558 | 204,328,971 | 96.50 | 76.69 |
| 6 | 175,971,560 | 170,976,906 | 97.16 | 79.96 |
| 7 | 199,418,705 | 192,886,854 | 96.72 | 75.95 |
| 8 | 210,669,584 | 205,154,195 | 97.38 | 81.64 |
| 9 | 276,214,801 | 267,270,128 | 96.76 | 78.33 |
| 10 | 246,151,052 | 238,036,360 | 96.70 | 79.58 |
| 11 | 223,362,488 | 216,354,286 | 96.86 | 76.41 |
| 12 | 188,697,992 | 182,975,778 | 96.97 | 78.40 |
| 13 | 218,035,022 | 210,387,481 | 96.49 | 77.00 |
| 14 | 245,754,296 | 239,530,090 | 97.47 | 82.67 |
| 15 | 255,874,670 | 247,226,074 | 96.62 | 78.61 |
| 16 | 215,533,526 | 209,652,147 | 97.27 | 79.77 |
