# Supplementary material for: Comparative genomics shows that viral integrations are abundant and express piRNAs in the arboviral vectors Aedes aegypti and Aedes albopictus
Source: BMC Genomics. 2017 Jul 5;18:512. doi: 10.1186/s12864-017-3903-3 (PMC5497376; doi:10.1186/s12864-017-3903-3)
Supplement: Supplementary file 5 — Viral integrations (NIRVS) in the Ae. aegypti and Ae. albopictus genomes. Classification was based on their sequence identity. Integrations with sequence identity >90% were grouped together. (DOCX 20 kb) [file 12864_2017_3903_MOESM5_ESM.docx]

**AdditionalFile5: Table S5.** **Viral integrations (NIRVS) in the *Ae. aegypti* and *Ae. albopictus* genomes.** Classification based on their sequence identity. Integrations with sequence identity > 90% were grouped together.

|  | *Aedes aegypti* NIRVS  (corresponding viral ORF) | *Aedes albopictus* NIRVS  (corresponding viral ORF) |
| --- | --- | --- |
| Unique F-NIRVS * | AeFlavi4, AeFlavi34,AeFlavi50, AeFlavi32, AeFlavi81, AeFlavi31, AeFlavi10, AeFlavi83, AeFlavi41 | AlbFlavi2, AlbFlavi36, AlbFlavi12_17, AlbFlavi6, AlbFlavi7, AlbFlavi10, AlbFlavi38, AlbFlavi1, AlbFlavi3, AlbFlavi4, AlbFlavi39, AlbFlavi40 |
| Unique R-NIRVS* | AeRha15, AeRha146, AeRha127, AeRha118, AeRha108, AeRha80, AeRha142, AeRha117, AeRha246, AeRha3, AeRha38, AeRha245, AeRha27, AeRha133, AeRha116, AeRha85, AeRha137, AeRha6, AeRha160, AeRha105, AeRha13, AeRha2, AeRha47, AeRha16, AeRha104, AeRha8, AeRha14, AeRha5, AeRha136, AeRha58, AeRha42, AeRha45, AeRha112, AeRha153, AeRha113, AeRha152, AeRha156, AeRha36, AeRha157, AeRha75B, AeRha77A | AlbRha79, AlbRha66, AlbRha45, AlbRha49, AlbRha1, AlbRha7, AlbRha88, AlbRha80, AlbRha2, AlbRha11, AlbRha74, AlbRha38, AlbRha44, AlbRha36, AlbRha42, AlbRha10, AlbRha14, AlbRha41, AlbRha4, AlbRha84, AlbRha73, AlbRha52, AlbRha3, AlbRha85, |
| F-NIRVS G2 | AeFlavi53, AeFlavi77 (NS1) | AlbFlavi8, AlbFlavi41 (NS1)** |
| F-NIRVS G3 | AeFlavi86, AeFlavi88, AeFlavi91, AeFlavi94, AeFlavi95, AeFlavi109, AeFlavi111, AeFlavi113, AeFlavi115, AeFlavi92 (NS1) | AlbFlavi23, AlbFlavi26, AlbFlavi37, AlbFlavi42, AlbFlavi22, AlbFlavi25 (NS1-NS2)** |
| F-NIRVS G4 | AeFlavi42, AeFlavi44, AeFlavi45, AeFlavi46, AeFlavi56, AeFlavi57, AeFlavi59, AeFlavi60, AeFlavi62, AeFlavi66, AeFlavi73, AeFlavi80 (NS3) | AlbFlavi32, AlbFlavi34, AlbFlavi31 (NS2)** |
| F-NIRVS G5 |  | AlbFlavi18, AlbFlavi27, AlbFlavi28, AlbFlavi20 (NS3)** |
| F-NIRVS G6 |  | AlbFlavi19, AlbFlavi24, AlbFlavi33 (NS1)** |
| R-NIRVS G2 | AeRha91, AeRha93 (G) | AlbRha71, AlbRha94 (N)** |
| R-NIRVS G3 | AeRha82, AeRha83 (G) | AlbRha48, AlbRha92 (N)** |
| R-NIRVS G4 | AeRha49, AeRha51 (N) | AlbRha32, AlbRha95 (G)** |
| R-NIRVS G5 | AeRha130, AeRha132 (N) | AlbRha58, AlbRha62 (G) |
| R-NIRVS G6 | AeRha55, AeRha154 (N)** | AlbRha18, AlbRha9, AlbRha28 (N)** |
| R-NIRVS G7 | AeRha54, AeRha125 (N)** | AlbRha83, AlbRha96, AlbRha87 (L)** |
| R-NIRVS G8 | AeRha11, AeRha9, AeRha33, AeRha72 (G)** | AlbRha12, AlbRha15 (N) |
| R-NIRVS G9 | AeRha98, AeRha100 (G) | AlbRha33, AlbRha43 (G)** |
| R-NIRVS G10 | AeRha110, AeRha111, AeRha138 (N)** |  |
| R-NIRVS G11 | AeRha75A, AeRha77B (G) |  |
| R-NIRVS G12 | AeRha162, AeRha163, AeRha147, AeRha96, AeRha102 (N)** |  |
| R-NIRVS G13 | AeRha57, AeRha169 (N)** |  |
| R-NIRVS G14 | AeRha143, AeRha144, AeRha89, AeRha87, AeRha149, AeRha148, AeRha150 (N)** |  |
| R-NIRVS G15 | AeRha168, AeRha61, AeRha66, AeRha71, AeRha70, AeRha63, AeRha68 (N)** |  |
| R-NIRVS G16a | AeRha28, AeRha30B (G) |  |
| R-NIRVS G16b | AeRha29, AeRha30A (G) |  |
| R-NIRVS G17 | AeRha139, AeRha123 (G)** |  |
