# Supplementary material for: Comparative genomics shows that viral integrations are abundant and express piRNAs in the arboviral vectors Aedes aegypti and Aedes albopictus
Source: BMC Genomics. 2017 Jul 5;18:512. doi: 10.1186/s12864-017-3903-3 (PMC5497376; doi:10.1186/s12864-017-3903-3)
Supplement: Supplementary file 6 — Details of AlbFlavi34. (DOCX 17 kb) [file 12864_2017_3903_MOESM6_ESM.docx]

**Additional File6: Supplemental File 1. Details of AlbFlavi34.**

ORF for NS3

**ORF for NS4**

**GC-AA breaking point as shown in figure 3, GGA-CC second breaking point**

**Short allele is comprised within the two breaking points**

**AlbFV34 primers for qRT-PCR -> expected** amplification product

>JXUM01S011498 dna:supercontig supercontig:AaloF1:JXUM01S011498:8671:11877

ATGAACTTGTGGATCATTCTTCAAACGGGGAGCTCAGCAGTGGTCCTGTTAGGATTCATGATCAGGCGCAAACTATCGATCGTCCTAGGCCATCAGCACCTAATGGCCTTGATCTGCCTCCAATTCCTTTTCTGGGTGGTGGAGCGACAACAACGCGCGTTTTCCATTTTCCTCGAAGCCGTAGCTGCGATAGTGCTCATCGGGGCATACCGCGGAATGACCCAGGACCTTCCCCCAGAGATCCTCGTATTCTGCCTCGTGATGGGGTGGAAGACAGCGTTAGCAATCGTTATCGTTGCCTTACTGATGCTCGGTTTGAACGCCTTTTACAAGTGGATGCTGAGCTTACAAGAGTCGAAAAGCTCCTTCAGGAACTCTGGCAAGAACGCTTGGTTCTGGATTGTTTCCTTCGCGAGCGCGGGGGCAATTTGGGCGGCGGAACGAGCCGAGCACCCGTCAGTGGCGGCAGTTCTCGCGCTCCTAACGATAGTTGCTTTCTTGTACATGGATCAAGCTAATGTGTCCATGGACTTGGAGTTCCTGTCCACAGGAGATATTCCCGAAGGAATTGCGCTTGAAGAAGACGAAGGAGGGAATTACCGGGATCTGCGCGGAACTTACAGTGAAGAAGGCATAACCATTGGTTCACCAACTGGAACAACCCAGATCCCTGAAACGATCGTCATCTTGTTGATCGGGTGTGCTCTAACGTCCGTTTCATTGTTCGTCGGGGCTTTGTATACAGTAATGGCTATCAGTACCAACATCCCACACAACCTCTACCGTATCTGTCGCTTAAAACTTAACGAGCATTGTAGATCCGACGACCTCCTAGGGTTTGGCGGAGCGGTTGCCCCCACACTAGAAACCAGTTTCGGAGACTTGCCCAATGGAGTTTATAGGATCATCGTGCGCAGCTTTATGGAAAACCGGCAACGCGGAATTGGTGTTGCAAAAAATGGGGTTTTTCACACCCTTATGCACGTCACCAGAGGAGAACCACTGAACTGGCGTGGCCGGCTCGTCTCTCTCCACTCAGGAAGCGCCCTTCGTGACGTCGTTTCGTATGGTGGGCCTTGGCAGTTGGACAGTCCCACTGTTGCAGATGAGGTGCTCCTCATGGCCTGCAAGCCCGACAAAACGGTTGAATACCACAAGTACAAACCCGGTGTGGTTAAAATCGACGATGAAACCGTCATGTTCATTAGCGTGGACTTTGGGAAGGGCTCTTCTGGCTCTCCTTTCTTCATTAATGGCGAAGTTGTTGGGTTTTACGGGTACGGGTTTTACATTGATGGCATATACCGGTCAATAGTTGCCGGTGGACGCCCAGGTGACGTTGTCACCAACGTGGTCGAGGATTCTACTCGCAAGTTTGTCACATGGCATCCTGGGAAGGGGAAAACTCGCAAGGTCATTGTTTCTGAAACCAAGGCAAATTTCGACTCTGGTTTACGCACCATTATCCTCACCCCCACGCGTGTGGTCATGGCTGAGGTGATTGATGCCCTTGCCGCGGTCGGCATAAACAGCGATCGGAACCTTATGTACTGCAAGAGGAACTTGGTAACTGTCGCGTGCCACGCAACATTTACGAAGTTCGTTCTGTCACATGGCGTCAAGAAGATTGGCGTGACTCTCATCATAATGGATGAATGTCATTTCATGGACCCAATGTCCATTGCCGCTCGTGGCATAATGGAACACCTGCACGAAAAGGGTACCAAGTTGATGTACCTGAGTGCTACTCCACCAGGACACACCCCAGATGGCGGTTCTAACTTCCCCATCCATGATCAGGCCATTGCTTTTCCCAGCTGGATGACTCCGGCTTGGATAAACGGTGTTAGGAAATCTCGCAACTCAAGAAAAGCAATCATGTTCGTCCCGTCCCATACGCAAGCCAACTATTTGGCTGGGTCCATACCTGGAGCTGTGTCGTTACACCGCGGAAATTTCTCCACAAACTATGCCCGCGCTGGTAGCGATGAAACCACCCTTGTGGTATCCACCGACATATCCGAGATGGGAGCGAACTTAGGAGTGGATATGGTCATTGACACCCGGAAGGTCTTGCGACCCATGGTCTTCTCGGAAAACCGCATCAAGTTGACGGAAACAGACGTCACGACGTCTTCTATGATTCAGCGCCGAGGACGCACCGGACGTCGAGCTCCGGGAAGCTACGTTTTCCCGGTAGACTGCCAAACAGAGGAGAACCCCGTCTCATGGTCCTGTTGGCCTGAGGCACAGATGCTGCTGGACCAGATGGGCATGACATTCATGCCAGAAGAAGCAACCTACAGCCAGCCTCCCGGTCGATACACCCTTGTTGGGGAGGACCTTATTAGGTTCATGAAGTTCCTGGACAAGGATGATATCCCAACCTGGCTGGCGTGGCATTGGGCTGAAGCTGCTGACCGCAGACACTCAGCCCTGTTCCAAGGAAACAGTACCGGACACATGCTAGACACTCGAT**ATGGAAGAATGGAATACAGACCTCAGTATGTTGACGACCGGTTTGAAAGACCGGTTTGAAAACATTGAATGGGATCAGCGAAAGTTGTCCATTGAGATGTATATCAATACCCGCAGCACTGCTAGTCTGTATGACATCCTCATGAGTGTTGACTGGCACGGAATCTGGAAAAGGACAGCCTCTTCTCTGTGGGACTTGCGTGACATCGTCAGTGGAGATCTACATGATCAGATCCTGACCGAACAGTTCCTGACTTCTGGAATGGCTTTTGTCCTAGGATGGGTCATAGCCATCGCGATTCTCCTCATTGTCTGGACTTTGGTCTGTCTGCTCTCCTACTCGCGCTCAGGAAAGAATTCCTATGAGCCCATGCCTGTATCGGACCCCTTGGGAGGAGGATTCGTGCTCACCTCCCCAAGCATACTACATTACTTCGGCGTTCCTTTGGGCTTTTGCGTGATAATCTTTCTGGCCATGTTCATTGTGTACCCGGTGTTGTACAAAGCCGCAGGGAATAGAAGCTACCTGGATAGTGACCTAGTAAAGTGGGTCATCATAGGATCATGTGTGATGTGCAGTGTTCTGGCTTGGGAAATGCGCCTGTTCCCAAATATTCGTGAGGACATAAAGAATGTCATGGCAGCCTCGGCTGCGCCAGTAGAAACCTCCACGCCATCTCCCCAGAACACCTGGTTTTCCCCCACTCCCTGGAATGGTGGTGTAAGATC**
