# Supplementary material for: Comparative genomics shows that viral integrations are abundant and express piRNAs in the arboviral vectors Aedes aegypti and Aedes albopictus
Source: BMC Genomics. 2017 Jul 5;18:512. doi: 10.1186/s12864-017-3903-3 (PMC5497376; doi:10.1186/s12864-017-3903-3)
Supplement: Supplementary file 8 — qRT-PCR based expression of AlbFlavi34 in different developmental stages of Ae. albopictus. (PDF 102 kb) [file 12864_2017_3903_MOESM8_ESM.pdf]

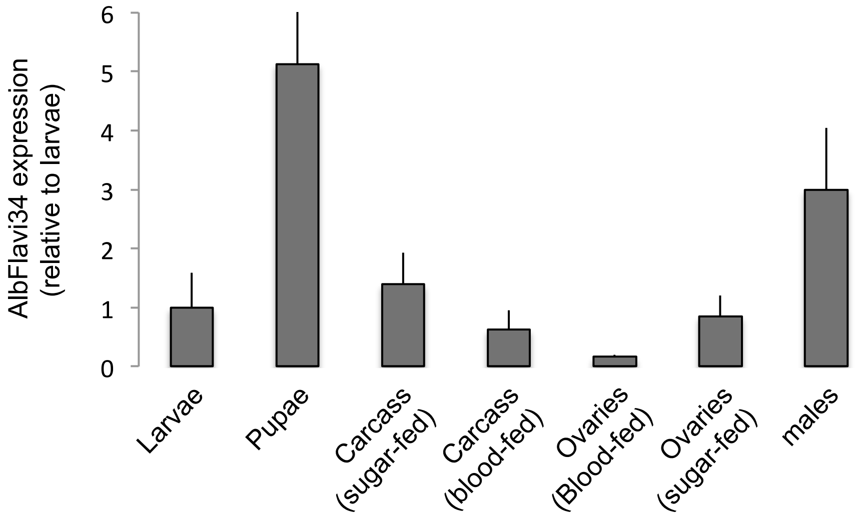

Additional File 8: Figure S1. qRT-based expression of AlbFlavi34 in different developmental stages of *Ae. albopictus*
